# Limited Variation in Codon Usage across Mitochondrial Genomes of Non-Biting Midges (Diptera: Chironomidae)

**DOI:** 10.3390/insects15100752

**Published:** 2024-09-28

**Authors:** Teng Lei, Xiaojun Zheng, Chao Song, Haobo Jin, Lingjun Chen, Xin Qi

**Affiliations:** 1 Zhejiang Provincial Key Laboratory of Plant Evolutionary Ecology and Conservation, School of Life Sciences, Taizhou University, Taizhou 318000, China; leiteng@tzc.edu.cn (T.L.); songchaonk@163.com (C.S.);; 2 Wenling Branch of Taizhou Ecological Environment Bureau, Wenling 317500, China

**Keywords:** Chironomidae, mitochondrial genome, codon usage

## Abstract

**Simple Summary:**

There are thousands of species of non-biting midges. They face various living pressures which may influence their codon usage in mitochondrial genomes. Codon usage analysis has been conducted on only a few species, and the codon usage across Chironomidae is still unclear. In this study, we sequenced a new mitochondrial genome and compared codon usage across as many genera as possible. Limited variation was observed in most genera, except one which presented a weaker codon usage bias. We speculated that most of its mitochondrial genes experienced natural selection. Additionally, three genes, *ND1*, *ND2* and *ND3*, were found to have experienced natural selection across most genera. To some extent, this work adds to our understanding of the evolution and phylogeny of non-biting midges.

**Abstract:**

The codon usage patterns of mitochondrial genomes offer insights into the evolutionary and phylogenetic studies of species. Codon usage analysis has been conducted in a few Chironomidae species, and the codon usage patterns in other species remain ambiguous. We aim to reveal the codon usage differences in the mitochondrial genomes across this family. We sequenced the first mitochondrial genome of the genus *Conchapelopia* and the third mitochondrial genome of the subfamily Tanypodinae. Then, we analyzed its relative synonymous codon usage and effective number of codons with registered mitochondrial genomes from 28 other genera. The results indicated that there was limited variation in codon usage across five subfamilies, Chironominae, Orthocladiinae, Diamesinae, Prodiamesinae and Tanypodinae. While *Parochlus steinenii* from Podonominae presented a weaker codon bias, *P. steinenii* possessed the most genes experiencing natural selection. Additionally, *ND1*, *ND2* and *ND3* were found to be the most frequently selected genes across all species. Our findings contribute to further understanding the evolutionary and phylogenetic relationships of Chironomidae.

## 1. Introduction

The Chironomidae consist of 11 subfamilies, over 400 genera and over 6000 species [1]. They colonize various habitats around the world and thus experience different living pressures, which may influence their evolutionary history [2]. Mitochondrial genomes are fundamental resources for the evolutionary and phylogenetic study of arthropods [3,4]. To date, Chironomidae mitochondrial genomes from at least 28 genera of six subfamilies have been reported. Chironominae and Orthocladiinae are the most diverse subfamilies, with mitochondrial genomes reported from ten and eight genera, respectively [5,6]. Mitochondrial genomes have been reported in five genera of Diamesinae [7]. Podonominae, Prodiamesinae and Tanypodinae have been reported with one, two and two mitochondrial genomes, respectively [8,9,10]. The remaining five subfamilies, Aphroteniinae, Buchonomyiinae, Chilenomyiinae, Telmatogetoninae and Usumbaromyiinae, have not been described with any mitochondrial genome.

Codon usage bias reflects the evolution of species or genes. Investigations of codon bias can reveal phylogenetic relationships between organisms as well as the molecular evolution of genes, and can identify selective forces that drive their evolution [11]. Relative synonymous codon usage (RSCU) analysis and effective number of codons (ENC) analysis on mitochondrial genomes have been applied to evaluate codon usage bias across three orders of aquatic insects, namely, Ephemeroptera, Plecoptera and Odonata [12]. Chironomidae comprise one of the most fundamental benthic insects, yet their mitochondrial codon bias is analyzed roughly in only a few studies. Fang et al. [5] found that ten Chironomidae and two Orthocladiinae species had similar characteristics of RSCU. Lin et al. [6] did not find a significant difference in RSCU among 12 Orthocladiinae sensu lato species. Cao et al. [13] revealed the absence of AGG, CGC, CUC and CUG codons in *Microtendipes umbrosus* mitochondrial genomes, and CGC was mostly lost in Chironominae and Tanypodinae. An ENC analysis performed on *M. umbrosus* revealed natural selection in mitochondrial genes, and it has not yet been performed on other Chironomidae species.

In this work, we sequenced the complete mitochondrial genome of *Conchapelopia togamaculosa*, a less studied species from Tanypodinae. We performed RSCU and ENC analyses on mitochondrial genomes across as many Chironomidae genera as possible, aiming to reveal the general characteristics of the codon usage patterns of this family.

## 2. Materials and Methods

### 2.1. Insects

Adults of non-biting midges were collected using light traps at Wencheng, Wenzhou, Zhejiang, China (27.649° N, 120.051° E) in May 2023. The specimens were preserved in 75% ethanol until taxonomic study and DNA extraction. A photograph of the specimen habitus was obtained with a DV500 5MP digital camera mounted on a Chongqing Optec SX680 stereomicroscope. Specimens were identified as *C. togamaculosa* according to the morphologic description by Niitsuma and Tang [14], and further confirmed using the DNA barcode of the *COI* gene. The nucleotide sequence of *COI* was obtained from the complete mitochondrial genome we assembled in this study. The *COI* sequence was matched to *C. togamaculosa* (Barcode Index Numbers ID: AAZ0260) in BOLD systems v4 with a similarity of 99.54%.

### 2.2. Mitochondrial Genome Sequencing of Conchapelopia togamaculosa

One male adult of *C. togamaculosa* was used for mitochondrial genome sequencing. Total genomic DNA was extracted from the abdomen using a DNeasy Blood & Tissue Kit (Qiagen, Hilden, Germany). The purified DNA was fragmented with sonication to a size of 350 bp and then sequenced on the Illumina NovaSeq 6000 platform with PE150 strategy. A total of 6.49 Gb clean reads were generated from raw reads using Trimmomatic ver. 0.40 [15]. Clean reads were assembled using NOVOPlasty ver. 4.3.1 with a K-mer of 39 bp [16]. The assembled sequence was annotated using MITOS2 [17] and tRNAscan-SE ver. 2.0.5 [18]. Coding regions were verified and manually corrected using MEGA X [19]. Moreover, samtools ver. 1.7 was used to calculate the depth per base [20]. GC content and GC skew were calculated using a window of 500 bp. The mitochondrial genome map, including genes, GC content, GC skew and sequencing depth per base, was visualized using Proksee at https://proksee.ca (accessed on 24 July 2024). The assembled mitochondrial genome and raw sequencing data were submitted to GenBank of NCBI.

### 2.3. Relative Synonymous Codon Usage Analysis of Chironomidae

There were at least 28 genera of Chironomidae with complete mitochondrial genomes available from GenBank of NCBI. In this study, we sequenced the first mitochondrial genome of the genus *Conchapelopia* and the third mitochondrial genome of the subfamily Tanypodinae. Relative synonymous codon usage was analyzed across 29 Chironomidae species listed in Table 1, each representing one genus. Mitochondrial protein coding sequences were downloaded from NCBI. Start codons, stop codons and internal amino acids were inspected for the accuracy of gene annotations. Gene sequences were submitted to MEGA X [19] to calculate the RSCU values [21]. These values were visualized using R package pheatmap ver. 1.0.12. The signatures of less frequently used codons (RSCU < 1) were covered by those of frequently used codons (RSCU > 1) in one heat map (Figure S1). Thus, we made two separate heat maps for the two datasets. In order to evaluate the similarity between the RSCU values across the specimens, specimens were automatically clustered when making the heat maps. A paired *t*-test was performed for the RSCU values between specimens after normality tests and variance homogeneity tests.

### 2.4. Effective Number of Codons Analysis of Chironomidae

We performed ENC analysis to evaluate codon usage bias across the 29 Chironomidae species. Lower ENC values implied a stronger codon usage bias. ENC values were calculated for every coding sequence, except *ATP8*, of every mitochondrial genome using CodonW ver. 1.4.2. *ATP8* was too short to be calculated. Because the specimens were all insects, before calculation, the genetic code was set as “Insects and Platyhelminthes Mitochondrial code”. GC3s, the GC content of the third synonymous position, was simultaneously calculated using CodonW ver. 1.4.2. If a gene underwent mutation but no selection, its expected ENC value (ENC_exp_) should be ENC_exp_ = 2 + GC3s + 29/[GC3s^2^ + (1 − GC3s)^2^] [29]. An observed ENC value (ENC_obs_) much lower than ENC_exp_ implied that the gene might have undergone selection.

## 3. Results

### 3.1. Mitochondrial Genome of C. togamaculosa

**Figure 1:**
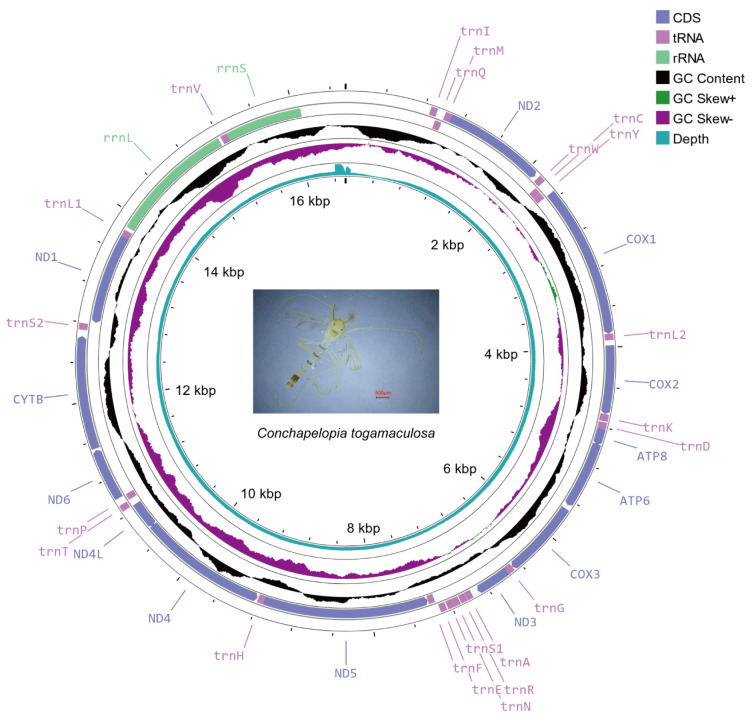
Circular map of *Conchapelopia togamaculosa* mitochondrial genome. From outer to inner rings: genes, GC content, GC skew, sequencing depth per base, and the scale of the genome. GC content and GC skew are both shown by 500 bp window and 1 bp step. A photo of *C. togamaculosa* is presented at the center of the map.

A circularized genome measuring 16,549 bp in length was obtained. Its average sequencing depth was 2473×, and the lowest depth per site was 661×. The genome composition (39.1% A, 37.3% T, 13.6% C, and 10.0% G) presented an A+T bias (76.4%), a positive AT skew (0.025) and a negative GC skew (−0.150). Through gene annotation, the genome was confirmed to be a mitochondrial genome of *C. togamaculosa*. The genome contained 37 genes, comprising 13 PCGs, 22 transfer RNA genes (tRNAs) and 2 ribosomal RNA genes (rRNAs) (Figure 1). The PCG region was 11,216 bp and comprised 67.8% of the genome. Gene arrangement stayed the same as for most other Chironomidae mitochondrial genomes, except *Stenochironomus*, the only genus found with mitogenomic gene rearrangement [26].

### 3.2. Relative Synonymous Codon Usage across Chironomidae

**Figure 2:**
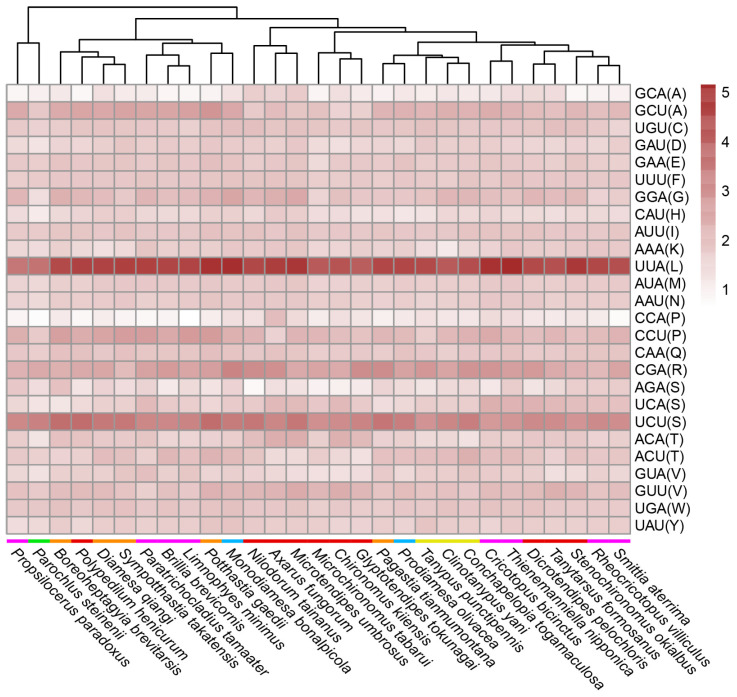
Relative synonymous codon usage (RSCU) of frequently used codons across Chironomidae species. RSCU values of frequently used codons (average RSCU value > 1) are shown in this heat map. Species are clustered automatically using R package pheatmap according to RSCU values. Colored bars in front of species names indicate subfamilies, purple for Orthocladiinae, green for Podonominae, orange for Diamesinae, red for Chironominae, blue for Prodiamesinae and yellow for Tanypodinae. The letters in parentheses after the codons are standard abbreviations for amino acids.

There were 26 frequently used codons encoding 20 amino acids in Chironomidae mitochondrial genomes. Fifteen amino acids, Cys (C), Asp (D), Glu (E), Phe (F), Gly (G), His (H), Ile (I), Lys (K), Leu (L), Met (M), Asn (N), Gln (Q), Arg (R), Trp (W) and Tyr (Y), all had their own unique, frequently used codons. The remaining five amino acids had more than one codon. Among them, Ala (A), Pro (P) and Ser (S) used the most frequent codons more than other codons. Lys (T) used ACA and ACU codons equivalently, and Val (V) used GUA and GUU codons equivalently (Figure 2). Through the analysis of frequently used codons, we did not find a clear correlation between RSCU and Chironomidae phylogeny. *C. togamaculosa*, *Clinotanypus yani* and *Tanypus punctipennis* all belonged to the subfamily Tanypodinae, and they formed one clade according to RSCU values. However, their RSCU values were not significantly different from those of other species, except *Microchironomus tabarui*, *Potthastia gaedii*, *Monodiamesa bonalpicola* and *Parochlus steinenii* (*p* < 0.05). Species from Chironominae, Orthocladiinae and Diamesinae were not clustered together individually. What drew our attention was the fact that *P. steinenii* from the subfamily Podonominae used codons with lower RSCU values than other species, implying a weaker codon usage bias. Its RSCU values differed significantly from those of all other species (*p* < 0.05) (Table S1).

**Figure 3:**
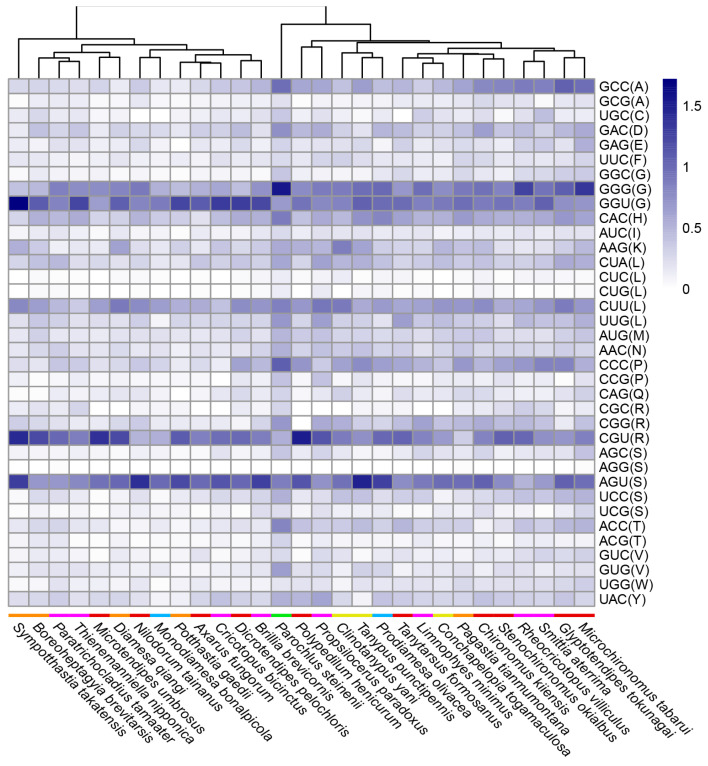
RSCU of less frequently used codons across Chironomidae species. RSCU values of less frequently used codons (average RSCU value < 1) are shown in this heat map. Species are clustered automatically by R package pheatmap according to RSCU values. Colored bars in front of species names indicate subfamilies: orange for Diamesinae, purple for Orthocladiinae, red for Chironominae, blue for Prodiamesinae, green for Podonominae, and yellow for Tanypodinae. The letters in parentheses after the codons are standard abbreviations for amino acids.

The remaining 36 codons encoding 20 amino acids were less frequently used. There was no obvious correlation between RSCU and Chironomidae phylogeny either. *C. togamaculosa* and two other Tanypodinae species formed two distinct clades. Interestingly, *P. steinenii* formed a unique clade, representing higher RSCU values than other species (Figure 3). *P. steinenii* was also the unique species whose RSCU values differed from all other species significantly (*p* < 0.05) (Table S2). Taken together, there was a weaker codon bias in the *P. steinenii* mitochondrial genome than other species. It remains unknown whether the weaker codon bias was a characteristic of *P. steinenii* alone or all the Podonominae species, because, to date, only the mitochondrial genome of this species has been documented within this subfamily.

### 3.3. Effective Number of Codons across Chironomidae

**Figure 4:**
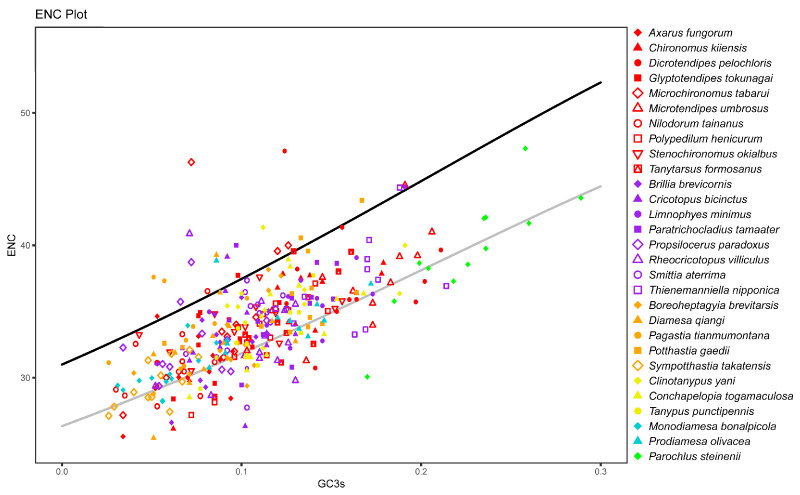
Effective number of codons (ENC) plot of mitochondrial genes. Mitochondrial coding sequences excluding *ATP8* are shown in the plot. The genes are plotted with GC3s, the GC content of the third synonymous position, in the x axis and with ENC in the y axis. Subfamilies are distinguished by the color of the dots, and species from the same subfamily are distinguished by shape. The black curve ENC_exp_ = 2 + GC3s + 29/(GC3s^2^ + [1 − GC3s]^2^) represents the relationship between expected ENC value (ENC_exp_) and GC3s under H_0_ (no selection). The gray curve corresponds to 85% of the black curve.

**Figure 5:**
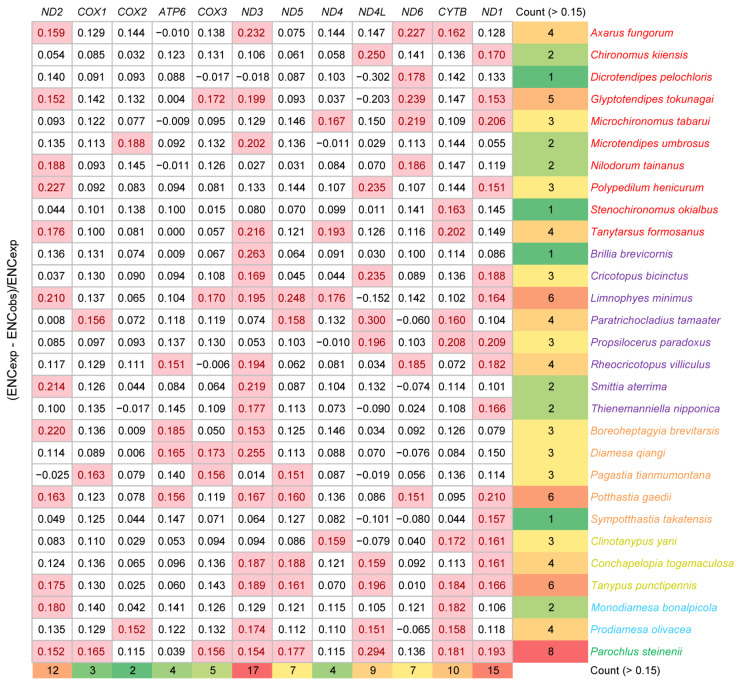
ENC ratio of mitochondrial genes. ENC ratio is calculated with (ENC_exp_ − ENC_obs_)/ENC_exp_. ENC ratios over 0.15 are highlighted with a pink background, indicating that the observed ENC values are less than 85% of ENC_exp_. Subfamilies are distinguished by the color of species names.

The ENC values of 12 mitochondrial coding sequences across all species were calculated. *P. steinenii* had the highest average ENC value (39.6). There was no significant difference in ENC values within other species. Their average ENC values ranged from 29.6 to 37.0. This result also supported the weaker codon bias in the *P. steinenii* mitochondrial genome. In the ENC plot, *P. steinenii* genes were distinguished from other species because of higher values of both ENC and GC3s (Figure 4). Most genes were distributed close to the curve of ENC_exp_, which meant that their codon bias mostly resulted from mutation. As we observed, a small portion of genes exhibited ENC values below 85% of ENC_exp_. Their codon bias might result from selection. Among these genes, eight belonged to *P. steinenii*, while other species possessed no more than six genes. From a genetic standpoint, *ND1*, *ND2* and *ND3* were the top three genes with ENC values below 85% of ENC_exp_. They appeared in 15, 12 and 17 species, respectively. Other genes appeared in no more than ten species (Figure 5). Additionally, the three genes had the lowest average ENC values across all species, implying a stronger codon bias.

## 4. Discussion

We enlarged the catalogue of Chironomidae species with complete mitochondrial genomes. The newly sequenced species, C. *togamaculosa*, belongs to the tribe Clinotanypodini and the subfamily Tanypodinae. Prior to this work, only two species in this subfamily, *Clinotanypus yani* (tribe Clinotanypodini) and *Tanypus punctipennis* (tribe Tanypodini), had been described with mitochondrial genomes [8,10]. Data of the new taxa contribute to learning the general characteristics of Chironomidae mitochondrial genomes. Undoubtedly, the catalogue is far from adequate to cover all Chironomidae species. Mitochondrial genomes have been reported in less than one in ten genera. This is partly because of the short history of Chironomidae mitochondrial genome studies. The first documented mitochondrial genome of the non-biting midge was that of *Chironomus tepperi*, which was reported in 2012 [30]. The remaining mitochondrial genomes have been reported mainly in the last four years. More mitochondrial genomes urgently need to be sequenced to resolve the evolutionary and phylogenetic relationships of Chironomidae.

The gene content of metazoan mitochondrial genomes is highly conserved [31]. All the registered mitochondrial genomes of non-biting midges, including C. *togamaculosa,* contain 13 PCGs, 22 tRNAs and 2 rRNAs. However, mitochondrial gene rearrangement is occasionally observed. Compared to vertebrates, Hexapoda exhibits a greater rate of gene rearrangement in different taxonomic orders [32]. The mitochondrial genomes of *Paracladura* (family Trichocera), *Culicoides* (family Ceratopogonidae), *Procontarina* (family Cecidomyiidae) and *Admontia* (family Tachinidae) have lost the ancestral dipteran gene arrangement [30,33,34,35]. In Chironomidae, *Stenochironomus* is the only genus found with gene arrangement [26]. However, *Stenochironomus* did not exhibit a unique codon usage pattern compared to its close relatives. Chironomidae exhibits a broadly conformable mitochondrial gene content and arrangement, and no relationships between codon usage bias and occasional rearrangement are observed, implying limited variation in genome content.

Limited variation in codon usage across the mitochondrial genomes of non-biting midges was observed. Similar codon usage patterns of mitochondrial genomes across the same family are observed in Dipteran Calliphoridae and Sarcophagidae. RSCU patterns among Calliphoridae, Sarcophagidae, Tachinidae, Agromyzidae and Culicidae are also similar [36]. In our results, *P. steinenii* uniquely presented a weaker codon bias. In Oestridae, *Gasterophilus pecorum* and *Gasterophilus intestinalis* also presented a more scattered RSCU pattern than other species, and this pattern was similar to *Bactrocera minax* and *Bactrocera umbrosa* (family Tephritidae) [36]. It remains unknown how these species evolved the scattered RSCU pattern. Codon usage bias evolved through mutation, natural selection, and genetic drift in various organisms [11]. Natural selection in non-biting midges is revealed using codon bias analysis [13]. *P. steinenii* is native to Antarctica and has a limited dispersal capability [37]. The distinct living pressure might have shaped its unique codon usage bias. Due to the limited catalogue of non-biting midges with registered mitochondrial genomes, we could not find a general rule for the weaker codon bias. We look forward to exploring this phenomenon with more mitochondrial genomes in the future.

NADH dehydrogenase plays an important role in cellular respiration. NADH dehydrogenase genes, especially *ND1*, *ND2* and *ND3*, are found to experience natural selection in most non-biting midges. In *Drosophila*, NADH dehydrogenase genes are found to accumulate many more amino-acid substitutions than cytochrome C oxidase genes [38]. *COX1* is a universal DNA barcode for delimiting species [39]. *ND1* exhibited higher diversity than *COI* in *Fasciola gigantica* [40]. NADH dehydrogenase genes have been applied to resolve the population diversity and phylogenetic relationships of mustelids, tapeworms, ricefishes, etc. [41,42,43]. The phylogeny of non-biting midges is ambiguous for some taxa. The NADH dehydrogenase genes or complete mitochondrial sequences are expected to clarify their phylogenetic status.

## 5. Conclusions

We provided the first mitochondrial genome of the genus *Conchapelopia* and the third of the subfamily Tanypodinae. There was no obvious variation in RSCU and ENC across Chironominae, Orthocladiinae, Diamesinae, Prodiamesinae and Tanypodinae species. *P. steinenii* from Podonominae presented a weaker codon bias, which might result from natural selection. *ND1*, *ND2* and *ND3* are the most frequently selected genes across all species.

## Figures and Tables

**Table 1:** List of species and GenBank accession numbers used in this study.

Subfamily	Species	GenBank Accession Number	Reference
Chironominae	*Axarus fungorum*	ON099430	[22]
Chironominae	*Chironomus kiiensis*	MZ150770	[23]
Chironominae	*Dicrotendipes pelochloris*	ON838257	[5]
Chironominae	*Glyptotendipes tokunagai*	MZ747091	[5]
Chironominae	*Microchironomus tabarui*	MZ261913	[24]
Chironominae	*Microtendipes umbrosus*	MZ981734	[13]
Chironominae	*Nilodorum tainanus*	ON838256	[5]
Chironominae	*Polypedilum henicurum*	MZ981735	[25]
Chironominae	*Stenochironomus okialbus*	OL753645	[26]
Chironominae	*Tanytarsus formosanus*	ON838255	[5]
Diamesinae	*Boreoheptagyia brevitarsis*	MZ043575	[7]
Diamesinae	*Diamesa qiangi*	MZ127839	[7]
Diamesinae	*Pagastia tianmumontana*	MZ231025	[7]
Diamesinae	*Potthastia gaedii*	OM302504	[7]
Diamesinae	*Sympotthastia takatensis*	MZ231026	[7]
Orthocladiinae	*Brillia brevicornis*	MZ424311	[6]
Orthocladiinae	*Cricotopus bicinctus*	OP006251	[27]
Orthocladiinae	*Limnophyes minimus*	MZ041033	[28]
Orthocladiinae	*Paratrichocladius tamaater*	MW837768	[6]
Orthocladiinae	*Propsilocerus paradoxus*	MW846254	[6]
Orthocladiinae	*Rheocricotopus villiculus*	MW373526	[8]
Orthocladiinae	*Smittia aterrima*	ON838254	[5]
Orthocladiinae	*Thienemanniella nipponica*	MZ424312	[6]
Podonominae	*Parochlus steinenii*	KT003702	[9]
Prodiamesinae	*Monodiamesa bonalpicola*	MW837770	[6]
Prodiamesinae	*Prodiamesa olivacea*	MW373525	[8]
Tanypodinae	*Clinotanypus yani*	MW373524	[8]
Tanypodinae	*Conchapelopia togamaculosa*	PP831866	This study
Tanypodinae	*Tanypus punctipennis*	MZ475054	[10]

## Data Availability

The data supporting the findings of this study are openly available in GenBank of NCBI at https://www.ncbi.nlm.nih.gov/ (accessed on 24 August 2024). The complete mitochondrial genome of *C. togamaculosa* has been released under accession number PP831866. The associated BioProject, BioSample and SRA accession numbers are PRJNA1160908, SAMN43763939 and SRR30664430.

## Supplementary Materials

The following supporting information can be downloaded at: https://www.mdpi.com/article/10.3390/insects15100752/s1, Figure S1: RSCU of mitochondrial codons across Chironomidae species. Colored bars in front of species names indicate subfamilies: red for Chironominae, purple for Orthocladiinae, orange for Diamesinae, yellow for Tanypodinae, blue for Prodiamesinae and green for Podonominae. The letters in parentheses after the codons are standard abbreviations for amino acids; Table S1: The *p*-value of paired *t*-test based on RSCU values of frequently used codons; Table S2: The *p*-value of paired *t*-test based on RSCU values of less frequently used codons.
