# Supplementary figures and images for: Limited Variation in Codon Usage across Mitochondrial Genomes of Non-Biting Midges (Diptera: Chironomidae)
Source: Insects. 2024 Sep 28;15(10):752. doi: 10.3390/insects15100752 (PMC11508583; doi:10.3390/insects15100752)

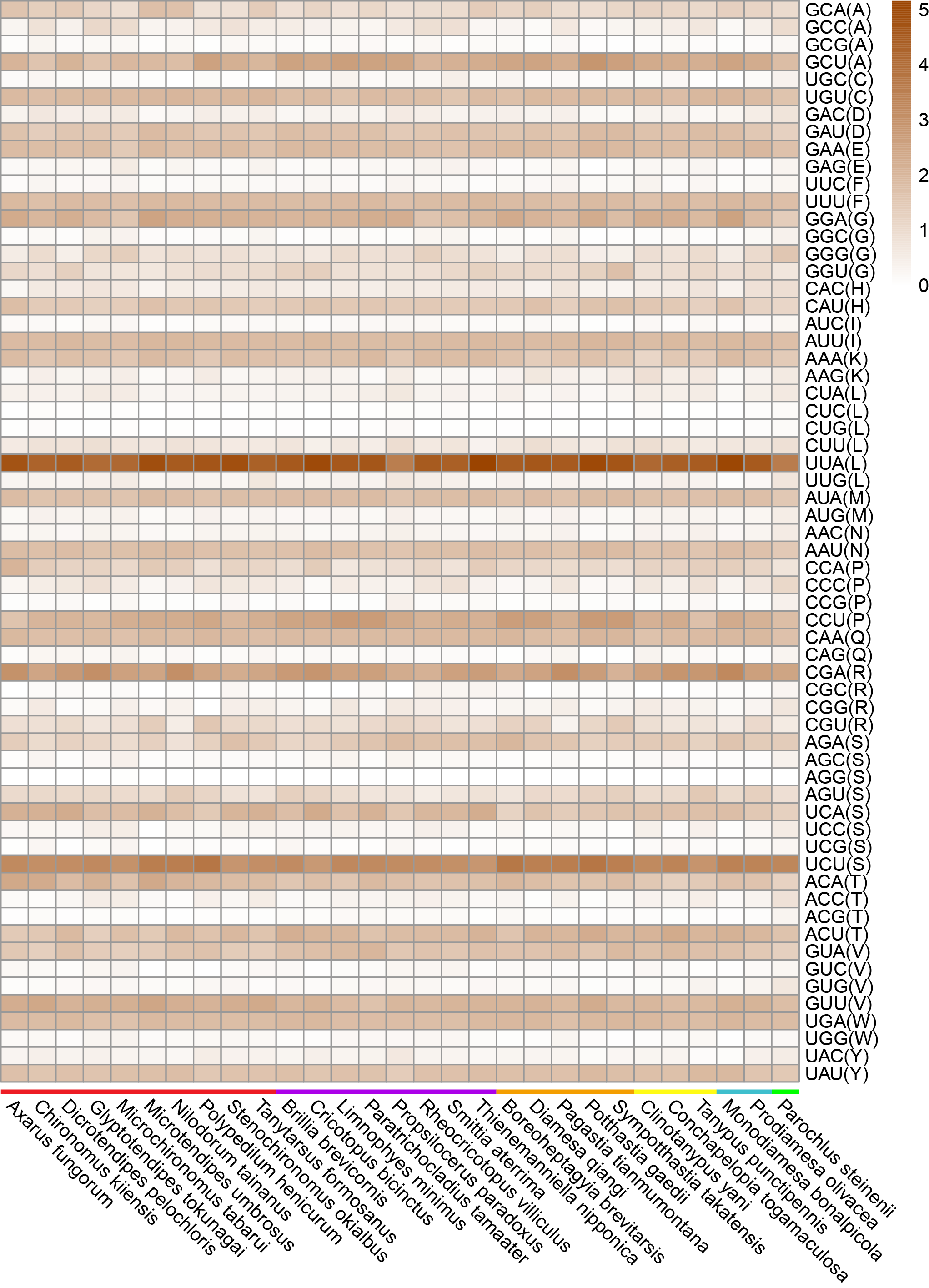

Supplement: Supplementary file 1 [file insects-15-00752-s001.zip › Figure S1.png]
